# Ewing's Sarcoma Disguised as Aneurysmal Bone Cyst Lesion: About a Case

**DOI:** 10.1155/2024/3549689

**Published:** 2024-04-02

**Authors:** Amine El Khassoui, Mariem Touraif, Driss Tahiri, El Mouhtadi Aghoutane, Tarik Salama, Redouane El Fezzazi

**Affiliations:** Pediatric Orthopedic Department, Mother and Child Unit, University Hospital Mohammed VI of Marrakech, Faculty of Medicine and Pharmacy, Research Laboratory: Childhood, Health and Development, Cadi Ayyad University, Marrakech, Morocco

## Abstract

Aneurysmal bone cysts are defined as benign lesions. They expose the patients to a higher risk of pathological fractures. The typical clinical and radiological aspects of the tumor usually do not require a pathological confirmation before a definite treatment. However, in some cases, a malignant tumor will have the same clinical and radiological characteristics of a begin lesion. Our case highlights this fact. We present a case of a 13-year-old patient that presented to us with a pathological fracture. The X-ray and CT scan were in favor of ABC; however, the postoperative pathology revealed an Ewing sarcoma. A salvage treatment became mandatory after this finding but was refused by the parents, resulting in the death of the patient 6 months later. A biopsy must be mandatory each time we have a suspicious aneurysmal bone cyst even with typical clinical and radiological characteristics before starting a treatment plan.

## 1. Introduction

Aneurysmal bone cysts (ABC) are defined as benign tumors that can become locally invasive. The rapid growth and the osteolytic capacity of ABC make the bone fragile and expose it to a higher risk of pathological fractures [1]. The diagnosis of aneurysmal bone cyst is mainly centered on the clinical and radiological aspects of the tumor; however, some malignant tumor can have a benign radiological appearance. A biopsy is needed in case of doubt or if a diagnosis challenge occurs [2]. Our study reports a case of a 13-year-old patient carrying an Ewing sarcoma misdiagnosed as an aneurysmal bone cyst of his right femur neck.

## 2. Case Report

**Figure 1:**
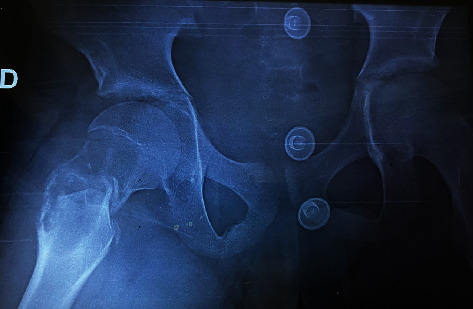
Femur neck fracture on a pathological bone (aneurysmal bone cyst).

**Figure 2:**
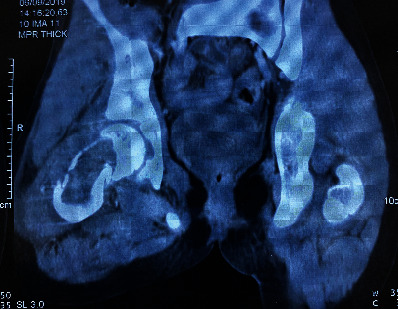
CT scan of the aneurysmal bone cyst.

**Figure 3:**
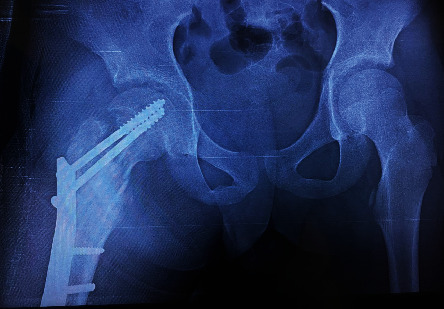
Postoperative X-ray after reduction of the fracture and curettage of the ABC.

Patient Y.T., aged 13, presented to our emergency department with an extreme pain in his right hip after a small trauma “minor fall to the ground.” First aid was administered upon admission, and then, a full-body examination was done showing impossible movements of the hip. An X-ray was performed (Figure 1) showing a suspected femur neck fracture on a pathological bone: aneurysmal bone cyst. CT scan of the hip was so performed, for better analysis of the lesion, confirming the diagnosis (Figure 2) with an HU value of 10 before injection and 15 after injection. Taking into consideration the clinical and radiological data that were typical, the diagnosis of a fracture on an aneurysmal bone cyst in the femur neck was established. Without an initial biopsy, we performed an open reduction and fixation of the fracture using osteosynthesis, after realizing a curettage and a bone graft mobilized from the homolateral tibia (Figure 3). During surgery, there was no suspicious aspect of the tumor or soft tissue lesion oriented towards a malignant tumor. Histology of the lesion revealed a malignant round cell tumor suggestive of an Ewing sarcoma that was confirmed by the immunohistochemistry study. The patient's family refused to carry out the treatment (a salvation amputation after rounds of chemotherapy) suggested by the orthopedist, pathology, and oncology teams, resulting into the patient's death 6 months after his initial diagnosis.

## 3. Discussion

ABC is a benign lesion that can be a locally aggressive tumor [3]. It presents many challenges in its diagnosis and treatment. The typical clinical and radiological aspects are usually the main diagnosis method. The X-ray shows an eccentric radiolucent lesion with expansile bone remodeling. A thin surrounding rim of the periosteum and subperiosteal bone is usually present. The cyst wall trabeculae impart the multilocular appearance. Occasionally, fluid-fluid levels are visible in CT scan, but the best imaging modality to identify the fluid-fluid levels is MRI. According to some authors [1], ABC are usually managed without the need of histological proof of its benign nature, although recently there are more and more case reports where a malignant lesion might present itself as benign clinical and radiological symptoms. Ewing's sarcoma is a malignant primary bone tumor [4]. It typically presents with an aggressive bone destruction and marrow permeation visible on radiographs and MRI [1, 5]. This typical aspect usually alarms the surgeon to the aggressive nature of the tumor, resulting to perform a biopsy before considering a definitive treatment. In rare cases, Ewing's sarcoma can disguise as a benign lesion. In our case, the patient presented with a typical clinical, radiological, and peroperatory symptoms in favor of an ABC. We have reviewed a study [2, 3] presenting the challenges in diagnosis and treatment of ABC, where 15 out of 25 patients that presented difficulties of diagnosis or treatment had no biopsy prior to a definitive surgery. This falls within our department-preferred method of treatment. When the patient shows typical signs of ABC, an initial biopsy before a definitive treatment is spared. Still, there have been rare reports of Ewing's sarcomas presenting as benign bone cyst [5, 6], and our case is one of them. The presence of these cases and the unfortunate outcomes of these tumors lead to reconsidering the way we approach benign-looking tumors and trigger a conversation on how and when we have to perform a biopsy before a definitive treatment of these benign-looking tumors, which might hide aggressive life-threatening malignant tumors. This case report changed our attitude towards patients carrying a suspicious ABC lesion. After this experience, our department's management of ABC has changed and all of them admit that they underwent an initial biopsy before a definitive treatment.

## 4. Conclusion

The diagnosis of aneurysmal bone cyst is often evident. The diagnosis is almost evident on the clinical and radiological exams. However, it is always necessary to keep in mind the probability of having a malignant tumor behind a benign-looking one, which should suggest that even evident benign radiological tumors should be biopsied to avoid undesirable surprises. A biopsy must be mandatory each time we have an aneurysmal bone cyst even with typical clinical and radiological characteristics before starting a treatment plan.

## Data Availability

The literature data supporting this case report are from previously reported studies and datasets, which have been cited. The processed data are available from the corresponding author upon request.
